# Supplementary material for: Feature selection by replicate reproducibility and non-redundancy
Source: Bioinformatics. 2024 Sep 10;40(9):btae548. doi: 10.1093/bioinformatics/btae548 (PMC11410923; doi:10.1093/bioinformatics/btae548)
Supplement: btae548_Supplementary_Data [file btae548_supplementary_data.zip › supplement.pdf]

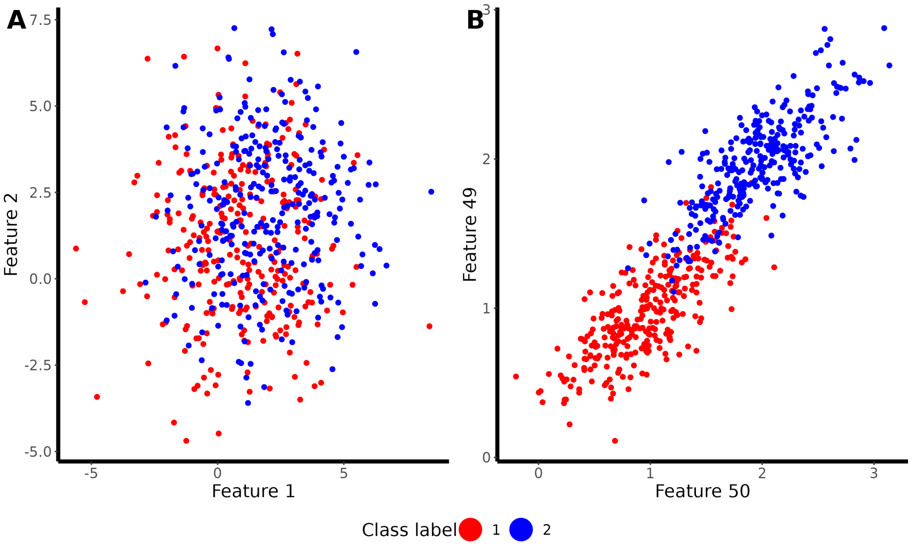

Figure S1: Scatter plots of simulated 2 class data. Features in (A) have lower variance than in (B) and show better class separation.

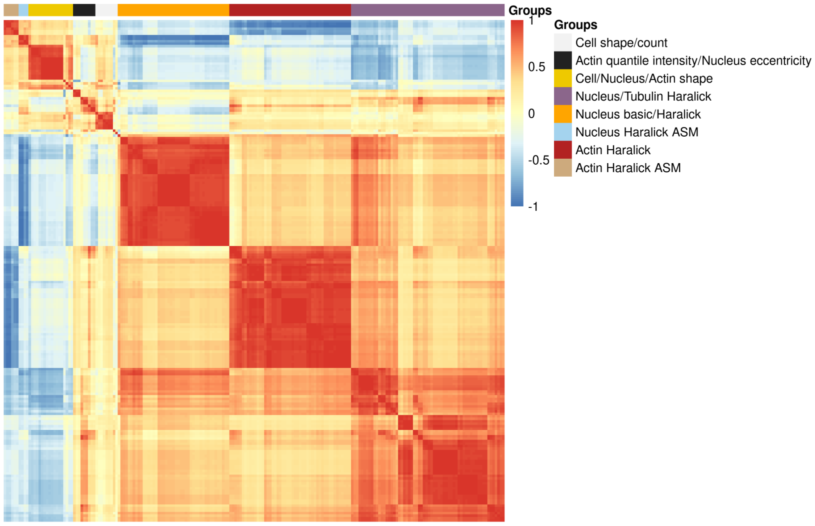

Figure S2: Correlation matrix of the features from (Laufer et al., 2013).

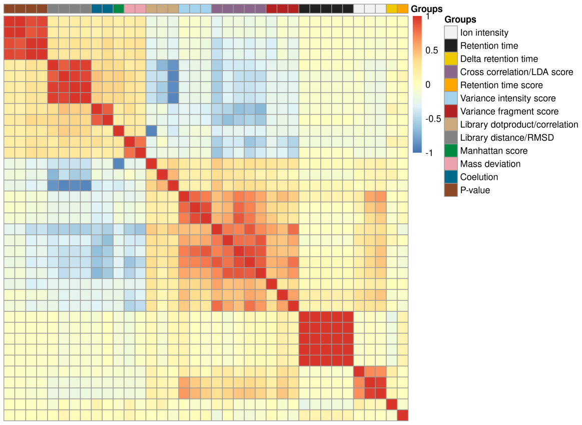

Figure S3: Correlation matrix of the features from (Collins et al., 2017).
