# Supplementary figures and images for: Feature selection by replicate reproducibility and non-redundancy
Source: Bioinformatics. 2024 Sep 10;40(9):btae548. doi: 10.1093/bioinformatics/btae548 (PMC11410923; doi:10.1093/bioinformatics/btae548)

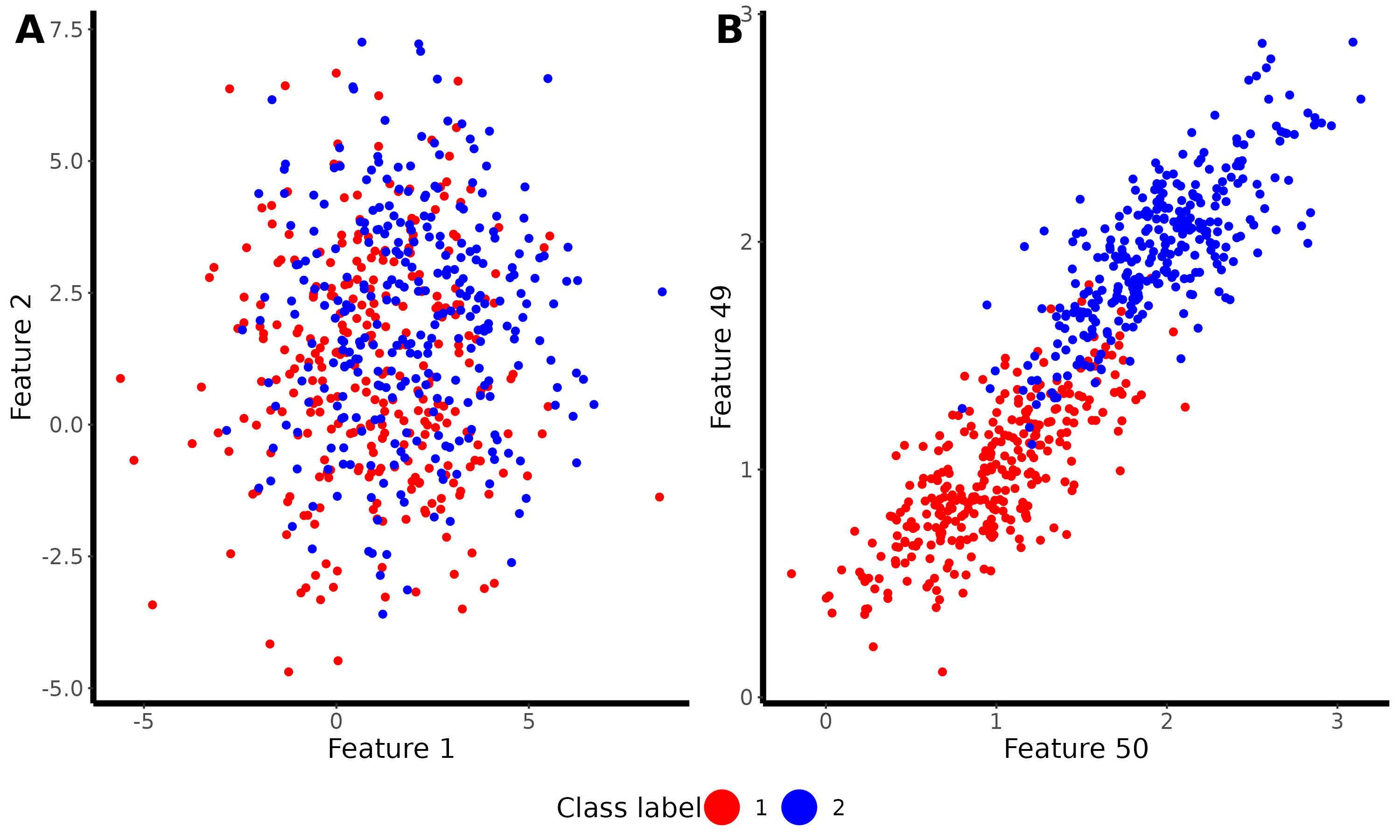

Supplement: btae548_Supplementary_Data [file btae548_supplementary_data.zip › FigureS1.png]

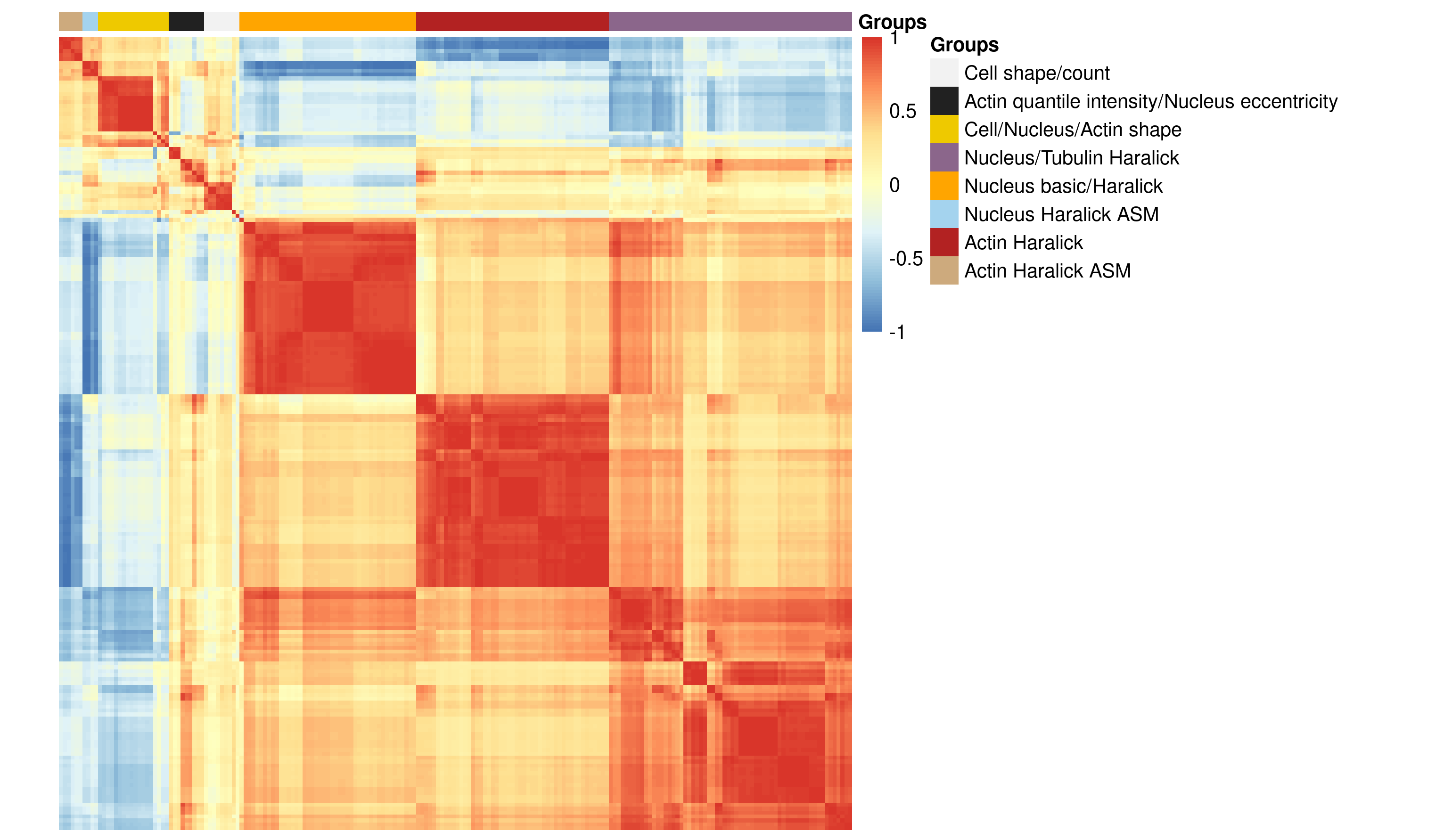

Supplement: btae548_Supplementary_Data [file btae548_supplementary_data.zip › FigureS2.png]

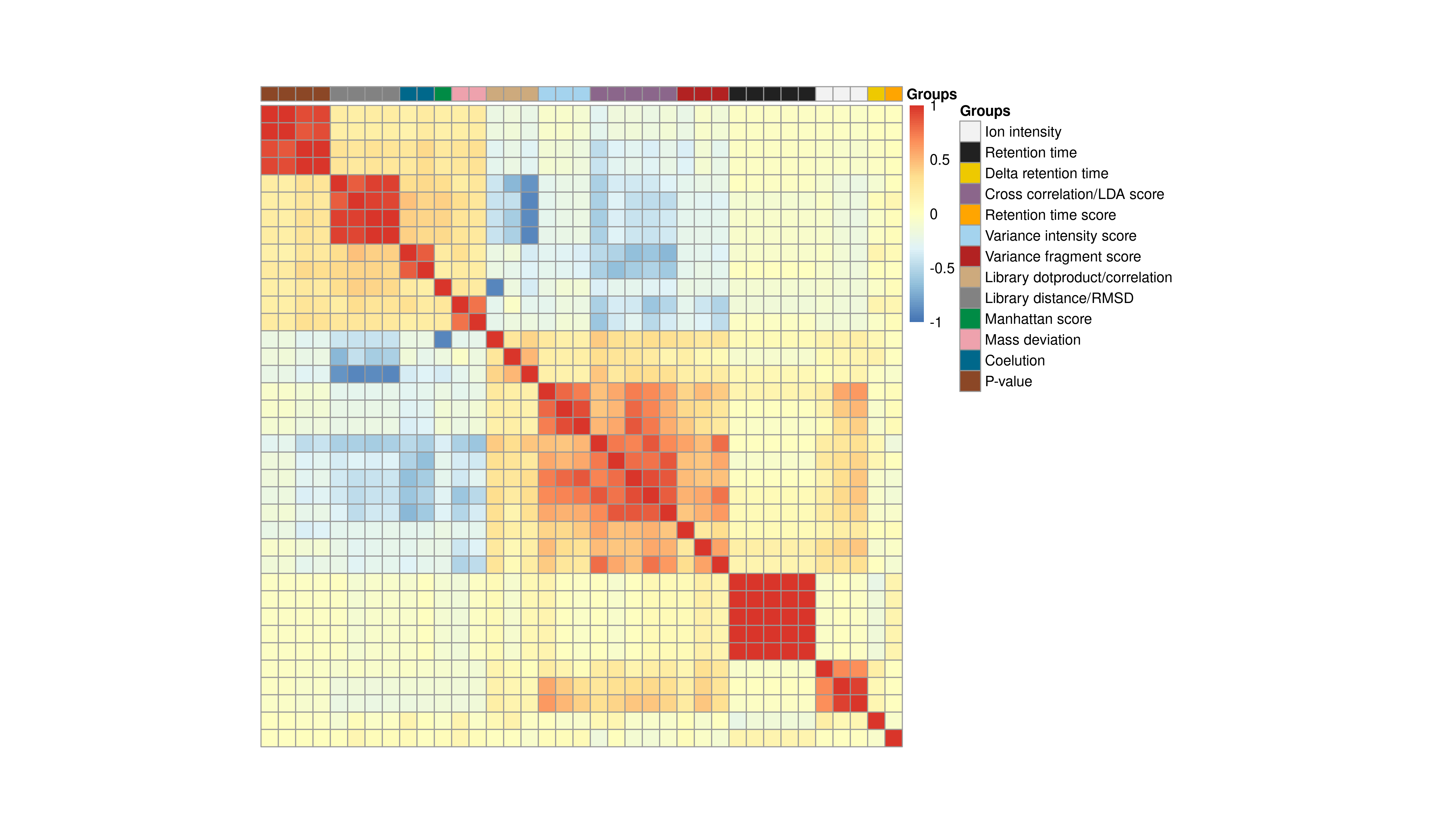

Supplement: btae548_Supplementary_Data [file btae548_supplementary_data.zip › FigureS3.png]
